# Detection of Microbial Translocation in HIV and SIV Infection Using the *Limulus* Amebocyte Lysate Assay is Masked by Serum and Plasma

**DOI:** 10.1371/journal.pone.0041258

**Published:** 2012-08-01

**Authors:** Ashwin Balagopal, Lucio Gama, Veronica Franco, Julia N. Russell, Jeffrey Quinn, Yvonne Higgins, Laura M. Smeaton, Janice E. Clements, David L. Thomas, Amita Gupta, for the NWCS 319 and ACTG 5175 study team

**Affiliations:** 1 Department of Medicine, Johns Hopkins University, Baltimore, Maryland, United States of America; 2 Department of Molecular and Comparative Pathobiology, Johns Hopkins University, Baltimore, Maryland, United States of America; 3 Center for Biostatistics in AIDS Research, Harvard School of Public Health, Boston, Massachusetts, United States of America; Karolinska Institutet, Sweden

## Abstract

**Objective:**

Microbial translocation (MT) is thought to be a major contributor to the pathogenesis of HIV-related immune activation, and circulating lipopolysaccharide (LPS) from Gram-negative bacteria is the principle measurement of this process. However, related research has been impeded by inconsistent LPS test results.

**Methods:**

Specimens were obtained from HIV-infected adults enrolled in the PEARLS study (ACTG A5175) and HIV-HCV co-infected participants enrolled in a study of liver disease staging using MRI elastography. Pig-tailed macaque specimens were obtained from SIV-infected and –uninfected animals. Samples were tested for LPS using the LAL assay with diazo-coupling modifications to improve sensitive detection.

**Results:**

When exogenous LPS was added to macaque plasma, >25% inhibition of LPS detection was found in 10/10 (100%) samples at 20% plasma concentration compared to control; in contrast 5/10 (50%) samples at 2% plasma concentration (p = 0.07) and 0/10 (0%) at 0.1% plasma concentration (*p = *0.004) showed >25% inhibition of LPS detection. Similarly, when LPS was added to human serum, >25% inhibition of LPS detection was found in 5/12 (42%) of samples at 2% serum concentration compared to control, while 0/12 (0%) of samples in 0.1% serum showed >25% inhibition of LPS detection (*p = *0.07). Likewise, LPS detection in human sera without exogenous LPS was improved by dilution: LPS was detected in 2/12 (17%) human samples in 2% serum, ranging from 3,436–4,736 pg/mL, compared to 9/12 (75%) samples in 0.1% serum, ranging from 123 pg/mL –60,131 pg/mL (p = 0.016). In a separate validation cohort of HIV-HCV co-infected participants sampled at two different times on the same day, LPS measured in 0.2% plasma and with diazo-coupling was closely correlated between the first and second samples (R = 0.66, p<0.05).

**Conclusions:**

Undiluted serum and plasma mask LPS detection. The extent of MT may be substantially underestimated.

## Introduction

Human immunodeficiency virus (HIV) progression to AIDS is associated with immune activation. [1] One proposed pathway for immune activation in HIV/AIDS is HIV-induced intestinal CD4+ T-cell depletion that alters intestinal immunity and enhances microbial translocation (MT) of commensal bacteria products (i.e. the “leaky gut” hypothesis). The cardinal expression of MT is blood lipopolysaccharide (LPS), the cell wall component of Gram-negative enteric organisms. Brenchley *et al.* found that persons with HIV infection and CD4+ T-lymphocyte depletion had higher levels of plasma LPS than controls using the *Limulus* Amebocyte Lysate (LAL) assay. [2] This finding was confirmed by some, but not all, studies of HIV and simian immunodeficiency virus (SIV) infection.[2]–[8] Inconsistencies in this literature do not appear to be explained by differences in the research subjects or study design, raising the hypothesis that limitations in the LPS assay itself might contribute.

Conventional LPS detection exploits an enzymatic clotting reaction in the horseshoe crab, *Limulus polyphemus.* The LAL assay couples the reaction with catalysis of a colorimetric substrate. The LAL assay is sensitive at detecting 1–10 pg/mL LPS, although this sensitivity is compromised by mammalian serum. [9], [10] In this study, we demonstrate LPS masking by serum and plasma from HIV- and SIV-infected hosts and uninfected controls using the LAL assay. We identified that LPS detection is only interpretable at concentrations far below the range typically used, a finding that has important implications for all LPS-related research including studies of the pathogenesis of HIV and SIV infection.

**Figure 1:**
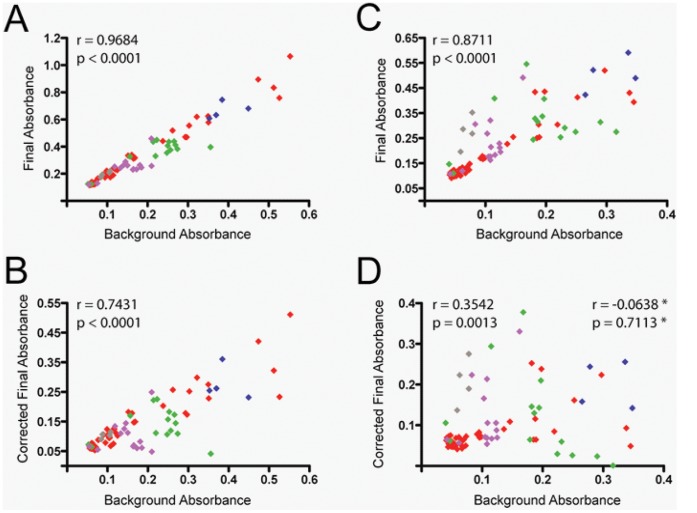
Correlation between background and final absorbance results (raw or corrected by background subtraction) in dilutions of SIV-infected and -uninfected macaque plasma samples tested for LPS content. Shown are bivariate analyses of background absorbance values and final absorbance (A and C) or corrected final absorbance (final absorbance minus background; B and D) read at their respective wavelengths: (A and B) final absorbance values were measured at 405 nm in the absence of the diazo-coupling modification or (C and D) at 540 nm after performing the diazo-coupling modification. Dilutions are color-coded (20% plasma concentration in red, 2% in blue, 1% in green, 0.5% in purple, 0.2% in gray). *Correlation and significance values marked with asterisks were calculated after removing all points representing 20% plasma (in red). Spearman’s correlation was used for all analyses.

## Methods

Plasma and serum were derived from three sources. The first source was plasma from 10 pigtailed macaques that were collected pre- and post-SIV infection (42 days post-infection) as part of ongoing studies of SIV progression and the use of antiretroviral therapy (ART). The second source was human serum obtained from the ACTG study A5175, a completed HIV treatment trial assessing responses to ART in diverse international settings. To validate that LPS masking is a generalized phenomenon in HIV infection, a third source of plasma was obtained from HIV-Hepatitis C virus (HIV-HCV) co-infected subjects who were recruited for a study of magnetic resonance elastography to determine liver disease stage. In this study plasma was obtained and tested for LPS at two different times during the same day in 10 subjects, before and after magnetic resonance elastography. All macaque and human specimens were stored at −80°C on-site or in the testing laboratory. Aliquots were thawed on the day of testing and tested in batch.

**Figure 2:**
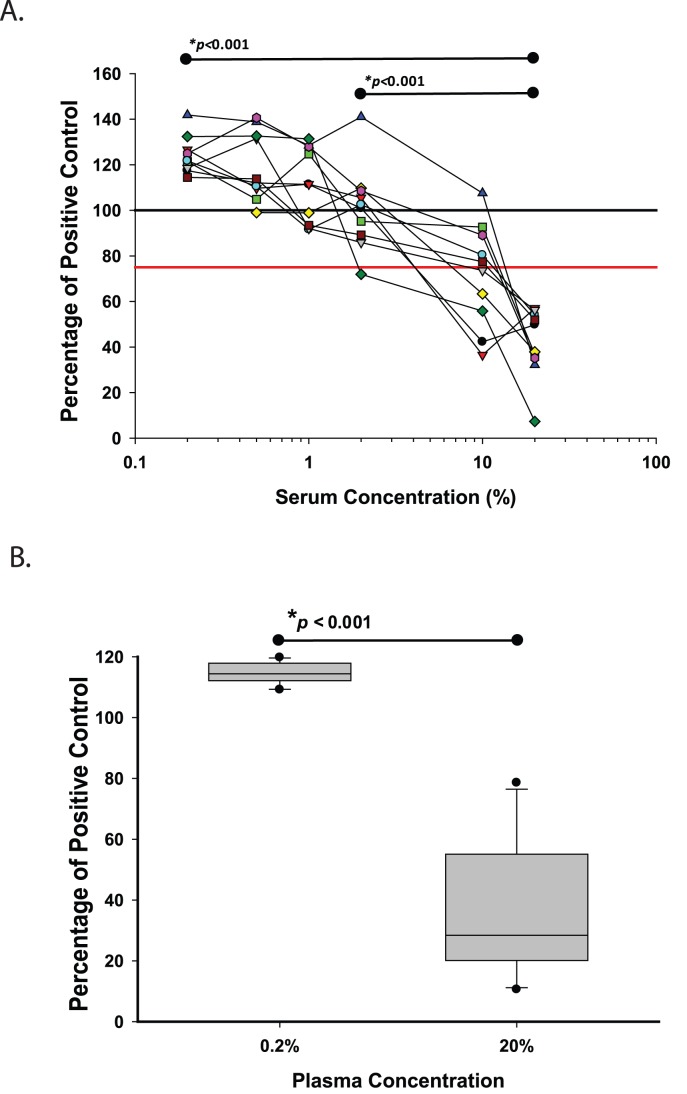
LPS masking in macaque plasma. Exogenous LPS was added to samples at a fixed concentration of LPS (50 pg/mL) from a reference *E. coli* standard after dilution in 10 mM MgCl_2_, as indicated. LAL testing with diazo-coupling was performed on all samples and each result was compared to an LPS standard at the same concentration in diluent alone. **A**) Plasma samples from 10 uninfected and SIV-infectemacaques were diluted from 20% (1∶5 dilution) to 0.2% (1∶500 dilution). Levels of no inhibition (black line) and 25% inhibition (red line) are indicated. **B**) Plasma samples from 10 SIV-infected macaques were diluted and measured in 20% and 0.2% concentration. Shown are mean ± SD for each animal. *Mean percent inhibition was compared between the indicated concentrations using the paired t-test.

**Table 1:** Characteristics of twelve randomly selected HIV-infected adult participants from the ACTG A5175 cohort and ten HIV-HCV co-infected adult participants from the Magnetic Resonance Elastography (MRE) study.

	ACTG 5175	MRE
	N = 12	N = 10
**Median Age, years (IQR)**	31.5 (27.5–35.3)	47.5 (44.5–52.3)
**Male, n (%)**	4 (33.3)	5 (50)
**Black, n (%)**	8 (66.7)	9 (90)
**On Antiretrovirals,** **n (%)**	7 (58.3)	10 (100)
**Median CD4+** **T lymphocytes/mm^3^ (IQR)**	244 (212–384)	269 (220–334)
**Median HIV-1** **RNA log_10_ copies/mL (IQR)**	2.6 (2.3–4.0)	Median <50
**Median Baseline ALT** **(U/L) (IQR)**	25 (20–30)	42 (36–69)
**HCV RNA log_10_ IU/mL**	NA	6.58 (5.84–6.76)

All MRE patients were HCV genotype 1; 2 (20%) had biopsy-proven cirrhosis.

LPS was measured using the LAL assay (LONZA, Walkersville, MD) as previously published but with the following modifications, unless otherwise stated. [3] A) Samples were diluted in LPS-free 10 mM MgCl_2_ (LONZA, Walkersville, MD) in series from 1∶5 (20% concentration) to 1∶1000 (0.1% concentration) and divided into two aliquots; a known amount of LPS (25 or 50 pg/mL) was added to the first tube (referred to as “exogenous LPS”), whereas none was added to the second (referred to as “naïve”). B) Samples were heated to 80°C for 12–15 minutes, transferred to LPS-free flat-bottomed 96-well plate (LONZA, Walkersville, MD) and kept at 37°C for testing. Background values were obtained at 405 and 540 nm. C) The assay was performed according to the manufacturer’s protocol but stopped by the addition of diazo-coupling reagents that derivatize p-nitroaniline, inducing a colorimetric change that is best detected at 540 nm (OD_540_). [11] Samples were then measured at 540 nm and LPS quantities were derived from a standard curve of known LPS concentrations. Spearman estimation was used to correlate the background and final absorbance. The proportion of inhibited samples at different concentrations was compared using McNemar’s exact test for non-parametric data, and paired t-tests were used to compare mean inhibition in the same samples at different concentrations.

### Ethics Statement

Study approval was obtained from the Institutional Review Boards (IRB) at sites of sample collection and testing (**Table S1**). Informed consent was obtained for the collection and storage of serum and plasma specimens from ACTG study A5175 participants and from magnetic resonance elastography study participants. In ACTG A5175, the Johns Hopkins University Institutional Review Board allowed a waiver for additional consent, since samples were de-identified, the research involved no more than minimal risk to subjects, and the waiver would not have adversely affect the rights and welfare of the subjects. Subjects in the magnetic resonance elastography study were specifically consented to measure LPS in their collected plasma. The study of SIV pathogenesis in pigtailed and rhesus monkeys was approved by the Johns Hopkins University Institutional Animal Care and Use Committee (IACUC). In the parent study, all efforts were made to limit the amount of pain and discomfort that these animals may have experienced during the course of infection. Appropriate medical and support care was provided as necessary. When invasive procedures were performed, intra- and post- procedural analgesics were administered along with antibiotics if needed.

**Figure 3:**
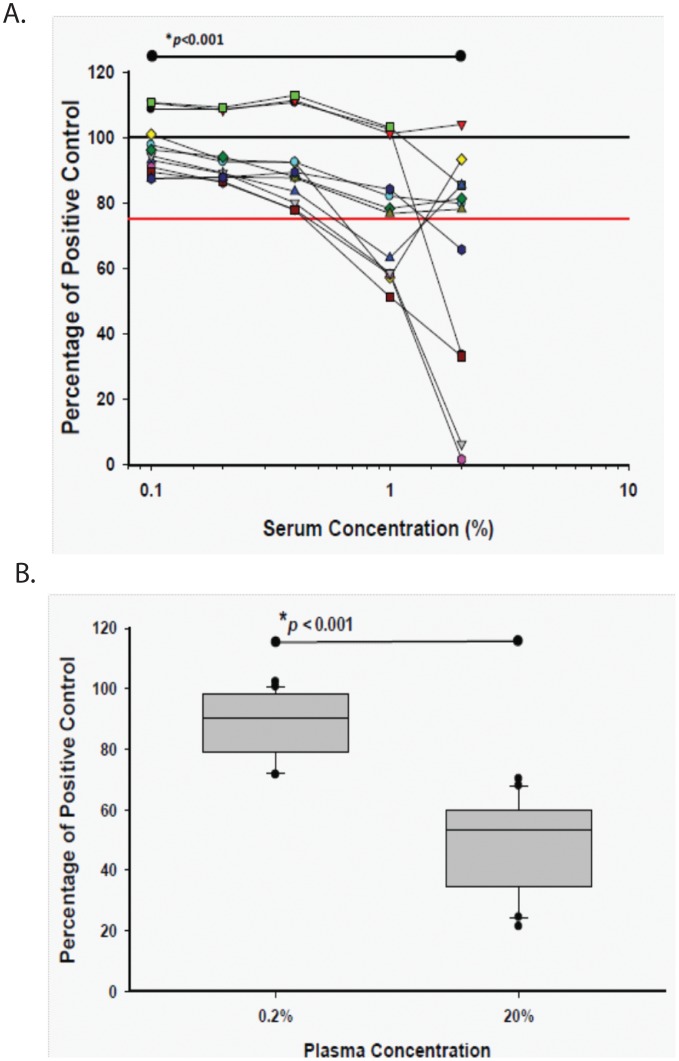
LPS masking in HIV-infected persons. **A**) Exogenous LPS of a fixed concentration from a reference *E. coli* standard was added to samples from 12 HIV-infected subjects that were diluted from 20% to 0.1% concentration. Levels of no inhibition (black line) and 25% inhibition (red line) are indicated. **B**) LPS was measured in each of 20 samples from from 10 HIV-HCV co-infected subjects and compared to a known quantity of LPS in assay diluent. The amount of inhibition was quantified for each specimen as a percent of the exogenously added LPS (100% indicates no assay inhibition) and compared in aggregate between the same samples in 0.2% plasma and 20% plasma. *Mean percent inhibition was compared between the indicated concentrations using the paired t-test.

**Figure 4:**
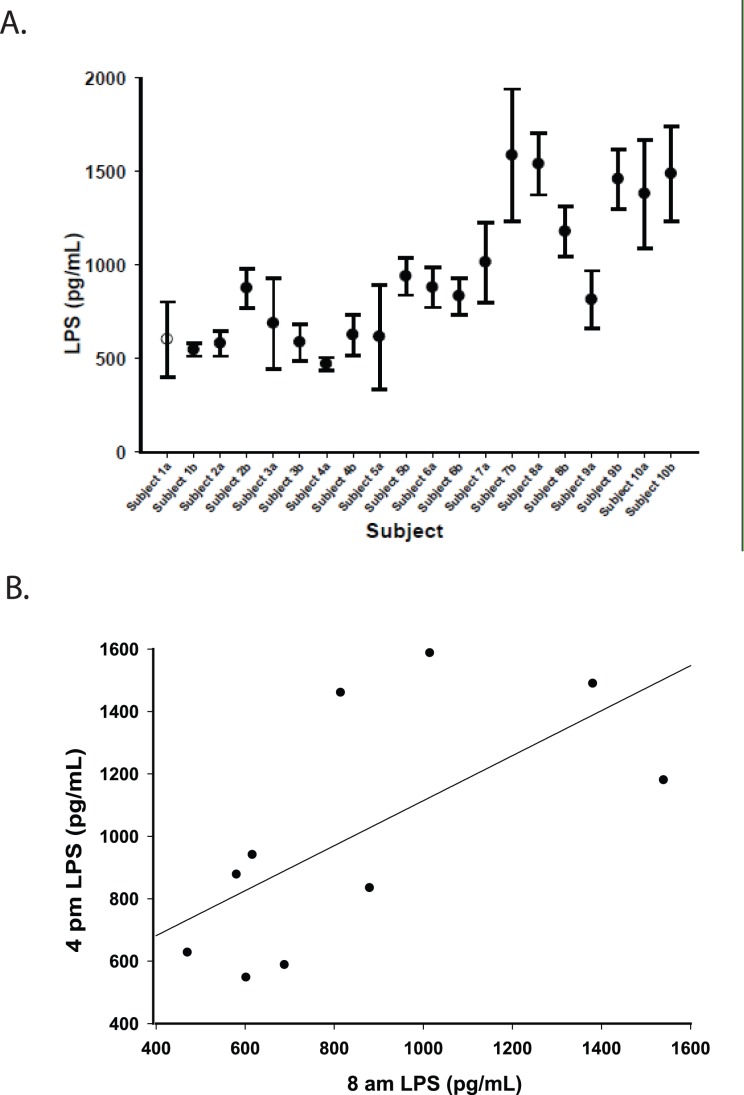
LPS measurements in 0.2% plasma are precise. A ) LPS was measured in triplicate (specimens 1–9) and duplicate (specimens 9, 10) in naïve samples diluted to 0.2% plasma from persons with HIV-HCV co-infection. Samples were obtained in the morning and afternoon for each subject and compared. Shown are mean ± SD for each subject at both time points, indicated by a subject number followed by “a” or “b” to denote the first and second time points, respectively. The SD for each sample ranged from 34 pg/mL to 354 pg/mL, and did not significantly differ between samples tested in triplicate compared to samples tested in duplicate. **B**) LPS was measured in samples from the same subject drawn at different times and correlated. Pearson R = 0.66, p<0.05.

## Results

### LPS Detection in Macaque Plasma Samples

All macaques were male with median age (IQR) of 4 (3–4.5) years**.** The median (IQR) CD4+ T-lymphocyte counts at the time of testing were 1394 (1086–1584) cells/µL for uninfected macaques and 373 (343–496) cells/µL after SIV infection. Median (IQR) SIV RNA for infected macaques at time of testing was 6.7 (5.8–7.3) log_10_ copies/ml. Initial LPS testing of SIV-uninfected and -infected plasma was performed in 20% plasma, as was described previously. [2] Visual inspection of the samples at this concentration revealed heightened turbidity. In the absence of diazo-coupling, the median background values were closely correlated to the absorbance values at the completion of the assay (r = 0.97, p<0.0001, **Fig. 1A**), and appeared to explain much of the corrected final absorbance values (r = 0.74, p<0.0001, **Fig. 1B**). Serially diluting each sample diminished some but not all background readings. After performing the diazo-coupling modification, the dependence of final on background absorbance values reduced in all plasma concentrations except the highest (20%), where it persisted (**Fig. 1C-D**): the correlation between background and corrected final absorbance values decreased when the measures in 20% plasma concentration were excluded from the analysis (r = 0.35, p = 0.0013 for all samples vs. r = 0.07, p = 0.71 for all samples excluding those measured in 20% plasma). The signal-to-noise ratio at this concentration masked detection of LPS in SIV-infected and -uninfected samples in spite of the diazo-coupling modification, prompting further study.

Exogenous LPS (50 pg/mL) was added to different concentrations of plasma (0.1% to 20%) from uninfected macaque and SIV-infected macaques, and compared to a control sample with the same amount of LPS added to diluent alone. Strikingly, 10/10 (100%) samples in 20% plasma had >25% lower OD_540_ values (>25% inhibition) compared to control (**Fig. 2A**). In contrast, 5/10 (50%) of samples in 10% plasma had >25% inhibition (*p* = 0.07), and 0/10 samples in 0.2% plasma showed >25% inhibition (*p* = 0.004). Importantly, relative differences in inhibition that were found between samples in 20% plasma were not preserved in 0.2% plasma. To validate that LPS masking in SIV infection can be generalized, plasma samples from 10 SIV-infected macaques were tested in a separate laboratory and with a second operator. Compared to a control sample with a known amount of LPS, 9 of 10 samples in 20% plasma had >25% inhibition; in contrast, 0 of 10 samples in 0.2% plasma had >25% inhibition (*p* = 0.008; **Fig. 2B**). Naïve samples (without exogenous LPS) were tested in triplicate for LPS and results were found to be consistently more sensitive at paradoxically lower concentrations: samples tested in 0.2% plasma had mean (SD) LPS concentrations of 633 (118) pg/mL, compared to the same samples tested in 20% plasma that had mean (SD) LPS concentrations of 18 (15) pg/mL (*p*<0.001, data not shown).

### LPS Detection in Human Serum Samples

Human serum samples from 12 HIV-infected adults were tested in parallel, and their characteristics are shown in **Table 1**. LPS was measured in samples tested in concentrations ranging from 2% and 0.1% serum (**Fig. 3A**). Mean (SD) LPS inhibition in 2% serum was 38% (35%), and ranged from −4% to 98%. Subjects with inhibition of LPS detection were indistinguishable from those without inhibition on the basis of their CD4+ T-cell count, HIV RNA level, or use of ART (data not shown). The mean (SD) inhibition of LPS detection improved to 3% (9%) in 0.1% serum and ranged from −11% to 13%. The proportion of samples with >25% inhibition decreased from 5 of 12 (42%) in 2% serum to 0 of 12 (0%) in 0.1% serum (p = 0.07); only 3 of 12 (25%) samples in 0.1% serum had >10% inhibition compared to 10 of 12 (83%) in 2% serum (p = 0.008). When naïve samples from the same HIV-infected individuals were diluted, a heightened sensitivity to detect LPS was observed; LPS was detected in 2 of 12 samples (17%) at 2% plasma, ranging from 3,436–4,736 pg/mL, and in 9 of 12 (75%) samples in 0.1% plasma, ranging from 123 pg/mL –60,131 pg/mL (p = 0.016).

### LPS Detection in Human Plasma Samples

To confirm that LPS masking is a general phenomenon in persons with HIV, and not limited to human serum, inhibition was validated in a separate group of 10 persons with HIV-HCV co-infection who were enrolled in a study of magnetic resonance elastography to measure the severity of liver disease. In this study, subjects had phlebotomy performed several times during the day, before and after elastography, from which plasma was collected. LPS was measured in 2 time-separated samples from each person, and testing was performed in 20% plasma and 0.2% plasma, with and without the addition of exogenous LPS. In samples in which exogenous LPS was added, the mean (SD) LPS inhibition in 20% plasma was 51% (16%) and ranged from 30% to 79%. In contrast, the mean (SD) LPS inhibition in 0.2% plasma was 12% (10%), and ranged from 2% to 28% in samples with exogenously added LPS (p<0.001**;** **Fig. 3B**). While LPS was not detectable in any naïve plasma sample tested in 20% plasma, LPS was detectable in all naïve plasma samples tested in 0.2% serum, and ranged from 471–1,586 pg/mL. In addition, replicate testing demonstrated that LPS detection was precise, both within a sample and between time points, for the same subjects (**Fig. 4A**). Notably, LPS measured from naïve samples in 0.2% plasma was closely correlated in the same subjects tested at two different times on the same day (R = 0.66, p<0.05; **Fig. 4B**).

### Conclusion

In this study we demonstrate that serum and plasma in HIV and SIV infection mask the detection of LPS using the LAL assay. Additionally, we report a method that enhances LPS detection. These results may have significant implications for studies of the pathogenesis of HIV infection and others for which LPS quantification is essential.

LPS is a heterogeneous collection of molecules that is found in the cell wall of most Gram-negative bacteria. [12] The fundamental structure of LPS, however, is conserved; it is composed of an inflammatory Lipid A domain, a core oligosaccharide, and a polysaccharide O-antigen. [13] Circulating LPS has been found in hosts with Gram-negative bacterial infections, and can act as a pathogen-associated molecular pattern that stimulates an innate immune response by binding to the Toll-like receptor 4 (TLR4) complex. [13] TLR4 binding triggers a downstream cascade that results in the up-regulation of pro-inflammatory cytokines such as tumor necrosis factor-α, and can be critical for priming an adaptive immune response. [12].

In HIV-infected patients there is mounting evidence that continued disease progression despite ART is due to systemic immune activation, a state wherein levels of inflammatory cytokines and markers of cellular activation are elevated. [1], [2], [14] Brenchley *et al.* linked intestinal CD4+ T-lymphocyte depletion in HIV infection to immune activation by demonstrating increased amounts of LPS circulating in individuals with chronic HIV infection and AIDS compared to uninfected persons and persons with early HIV-1 infection; the authors found similar evidence of MT in SIV-infected Rhesus macaques. [2] In contrast, Redd *et al.* did not find any association of LPS levels with AIDS progression in a longitudinal African cohort of HIV-infected persons, despite the presence in that cohort of persons with rapid disease progression. [4] Understanding these discordant results will require further testing, but already concerns have been raised that the conventional detection of LPS may lead to inconsistent results. [6] Our findings illustrate the difficulty in measuring LPS in HIV and SIV infection unless LPS masking is considered.

Inhibition of LPS detection has been described previously. [9], [10] While attempts have been made to identify the assay-inhibiting agent, none have been revealing. It is clear, however, that LPS recovery from serum and plasma can be improved at paradoxically low sample concentrations. Past reports have demonstrated that 1–10% serum concentrations have been sufficient to mitigate LAL inhibition, but those studies were not performed in HIV or SIV-infected samples. [9], [10] In the present study inhibition was diversely found in macaques and in humans, and concentrations as low as 0.2% were required for reliable quantification of LPS in serum and plasma. In contrast, recent studies of LPS detection in HIV or SIV-infected samples have used serum and plasma concentrations of up to 20% (1∶5 dilution) and did not assess for inhibition.[2]–[4] Although dilution of samples to 0.1% concentration reduces the lower limit of detection, this is balanced by the increased sensitivity of detection. In this study, serum and plasma dilution improved both the number of samples with detectable LPS and the total amount of LPS found in each sample.

Relative differences between LPS measurements might still be valid if inhibition occurred to the same degree between samples at a dilution, but are less interpretable if the amount of inhibition was specimen-dependent. In this study we found that the range of LPS inhibition in human serum varied from −4% to 98% at higher concentrations, but decreased to −11% to 13% at concentrations <0.5%, and similar results were found in plasma (**Fig. 3**). These findings suggest that relative differences in LPS detection at higher serum and plasma concentrations cannot be used as surrogates for LPS amounts observed at lower concentrations.

There are a variety of methods used to minimize LAL inhibition, including heating samples before assay performance as was done in our study. In addition, chloroform-based serum extraction can reduce the amount of inhibition if sufficient starting material is available. Diazo-coupling, specimen heating, and sample dilution attenuated LPS masking inhibition in this study.

In this report we illustrate that serum and plasma from hosts with HIV or SIV infection mask the detection of LPS more than has been previously described in uninfected persons. Therefore it is likely that earlier studies may have underestimated microbial translocation. We recommend that future studies of microbial translocation in HIV and SIV infection use a combination of techniques to minimize LPS masking, including tested diluted samples and employing diazo-coupling modifications.

## Supporting Information

Table S1 **International Testing Sites for ACTG study A5175.** Shown is the full list of each testing site and its associated IRB that was responsible for approving the use of participants’ samples for study.
(DOCX)
