# Supplementary material for: Detection of Microbial Translocation in HIV and SIV Infection Using the Limulus Amebocyte Lysate Assay is Masked by Serum and Plasma
Source: PLoS One. 2012 Aug 1;7(8):e41258. doi: 10.1371/journal.pone.0041258 (PMC3409852; doi:10.1371/journal.pone.0041258)
Supplement: Table S1 — International Testing Sites for ACTG study A5175. Shown is the full list of each testing site and its associated IRB that was responsible for approving the use of participants’ samples for study. (DOCX) [file pone.0041258.s001.docx]

**Table S1.**

Institution Name: Asociacion Civil Impacta Salud y Educacion - Miraflores, CRS

IRB: Asociacion Civil Impacta Salud y Educacion Institutional Review Board

Institution Name: Beth Israel Medical Center

IRB: Beth Israel Medical Center Institutional Review Board

Institution Name: College of Health Sciences, University of Zimbabwe, Parirenyatwas CRS

IRB: Medical Research Council of Zimbabwe

Institution Name: Columbia University Medical Center

IRB: Columbia University Medical Center Institutional Review Board

## Institution Name: Cook County Hospital Core Center

IRB: John H Stoger Hospital Cook County Institutional Review Board

Institution Name: Duke University Medical Center

IRB: Duke University Institutional Review Board

Institution Name: Harbor-UCLA Medical Center

IRB: Los Angeles Biomedical Research Institute Human Subjects Committee

Institution Name: Hospital Nossa Senhora da Conceição, Porto Alegre

IRB: Comitê de Ética em Pesquisa do Grupo Hospitalar Conceição

Institution Name:  Instituto de Pesquisa Clinica Evandro Chagas-FIOCRUZ (IPEC-FIOCRUZ)

IRB: Instituto de Pesquisa Clinica Evandro Chagas Ethics Review Board

Institution Name: Les Centres GHESKIO

IRB: Gheskio Centers Institutional Review Board - Comite des Droits Humains

Institution Name: Malawi College of Medicine-Johns Hopkins Research Project

IRB: In Malawi: College of Medicine Research Ethics Committee (COMREC)

In the US: Johns Hopkins Bloomberg School of Public Health Institutional Review Board (JHSPH IRB)

Institution Name: New York University School of Medicine

IRB: The Institutional Review Board (IRB) at the New York University School of Medicine (SoM)

Institution Name: Northwestern University

IRB:  Northwestern University Office for the Protection of Research Subjects

Institution Name: Research Institute for Health Sciences, Chiang Mai

University

IRB: 1. Human Experimentation Committee, Research Institute for Health Sciences RIHES), Chiang Mai University, Chiang Mai, Thailand; 2. Research Ethics Committee, Faculty of Medicine, Chiang Mai University, Chiang Mai, Thailand; 3. The Ethical Review Committee for Research in Human Subjects, Ministry of Public Health, Thailand

Institution Name: Rush University Medical Center

IRB: Rush University Medical Center Institutional Review Board

Institution Name:  The Ohio State University Medical Center

IRB: The Office of Responsible Research Practices; OSU Biomedical Sciences Institutional Review Board

Institution Name: The Miriam Hospital, affiliate of the Alpert Medical School of Brown University

IRB: Clinical Research Review Board,The Miriam Hospital

Institution Name: UC Davis School of Medicine

IRB: UC-Davis Institutional Review Board

Institution Name UCLA CARE Center

IRB: Office of the Human Research Protection Program UCLA Medical Institution Review Board

Institution Name: University of Cincinnati ACTU

IRB: University of Cincinnati IRB

Institution Name: University of Colorado Denver

IRB: Colorado Multiple Institutional Review Board

Institution Name:   University of Hawaii

Name of Ethics Committee/Institutional Review Board: University of Hawaii Committee on Human Subjects

Institution Name: **University of KwaZulu Natal - Durban International Clinical Trials Unit (UKZN)**

IRB: **UKZN Biomedical Research Ethics**

Institution Name: University of Minnesota

IRB: University of Minnesota Institutional Review Board

Institution Name: University of North Carolina

IRB: UNC Office of Human Research Ethics

Institution Name:  University of North Carolina Project, Kamuzu Central Hospital

IRB:  National Health Sciences Research Committee (Malawi) and the University of North Carolina School of Medicine Protection of Human Subjects Committee

Institution Name:  University of Pennsylvania

IRB:  University of Pennsylvania Institutional Review Board

Institution Name: University of Rochester

IRB: University of Rochester Institutional Review Board

Institution Name: University of Southern California

IRB: University of Southern California Health Science Center Los Angeles Institutional Review Board

Institution Name: University of the Witwatersrand

IRB: University of the Witwatersrand Human Research Ethics Committee

Institution Name: UT Southwestern Medical Center at Dallas

IRB: UT Southwestern Medical Center Institutional Review Board

Institution Name: UTMB at Galveston

IRB: The University of Texas Medical Branch at Galveston Institutional Review Board

Institution Name: Vanderbilt University Medical Center

IRB: Human Research Protection Program

Institution Name: Washington University School of Medicine

IRB: Washington University Human Research Protection Office

Institution Name: Weill Cornell Medical College

IRB: Weill Cornell Medical College Institutional Review Board
